# Analysis of Cardiac Computed Tomography: Investigating the Relationship Between Coronary Microvascular Dysfunction and Left Heart Remodeling in Patients With Myocardial Ischemia Due to Non-Obstructive Coronary Artery Disease

**DOI:** 10.31083/RCM49529

**Published:** 2026-07-17

**Authors:** Yanli Yu, Xincheng Li, Linlin Sun, Haipeng Liu, Xinhong Wang, Zhen Wang

**Affiliations:** ^1^The Fourth School of Clinical Medicine, Zhejiang Chinese Medical University, Hangzhou First People's Hospital, 310053 Hangzhou, Zhejiang, China; ^2^Department of Radiology, Affiliated Hangzhou First People's Hospital, School of Medicine, Westlake University, 310006 Hangzhou, Zhejiang, China; ^3^Centre for Technology Transfer, Universidad Santa Paula, Curridabat, 11803 San José, Costa Rica; ^4^National Medical Research Association, Leicester, UK; ^5^Cardiovascular Analytics Group, Hong Kong SAR, China; ^6^Department of Radiology, The Second Affiliated Hospital Zhejiang University School of Medicine, 310009 Hangzhou, Zhejiang, China

**Keywords:** X-Ray computed, coronary microvascular dysfunction, myocardial ischemia, tomography, ventricular remodeling

## Abstract

**Background:**

Coronary microvascular dysfunction (CMD) represents a key pathological mechanism in patients with ischemia and non-obstructive coronary artery disease (INOCA). Structural and functional abnormalities of the left heart are associated with poor prognosis. The aim of this study was to evaluate the imaging features of early left-heart remodeling associated with CMD in patients with INOCA, including structural and functional changes.

**Methods:**

A total of 74 patients underwent coronary computed tomography angiography (CCTA) and invasive physiological assessment. They were classified into two groups: the CMD group (index of microcirculatory resistance (IMR) >25 or coronary flow reserve (CFR) <2.0, n = 36) and the control group (n = 38). Structural parameters included left ventricular mass (LVM), left ventricular mid-diastolic volume (LVMDV), left ventricular mid-systolic volume (LVMSV), left atrial mid-diastolic volume (LAMDV), and left atrial mid-systolic volume (LAMSV), all of which were indexed to body surface area (LVMi, LVMDVi, LVMSVi, LAMDVi, and LAMSVi, respectively). The concentricity index (CI) and left ventricular mid-diastolic wall thickness (LVMDWT) were also evaluated. Functional parameters included LAMDV, LAMSV, the diastolic expansion (DE) index, and left ventricular ejection fraction (LVEF).

**Results:**

The CMD group showed significantly higher values for LVMi, LVMDVi, LVMSV, LVMSVi, LAMDV, LAMDVi, LAMSV, and LAMSVi than the control group (*p *= 0.023, 0.018, 0.025, 0.017, 0.003, 0.002, <0.001, <0.001, respectively). No significant differences were found in the absolute values of LVM, LVMDV, CI, LVMDWT, DE, or LVEF (*p *= 0.113, 0.080, 0.868, 0.879, 0.406, 0.289, respectively). Multivariable logistic regression analysis revealed that higher LAMSVi (adjusted odds ratio (OR) [AOR] = 1.129, 95% confidence interval (CI) 1.052–1.211, *p *< 0.001), LAMDVi (AOR = 1.133, 95% CI 1.048–1.225, *p *= 0.002), LVMi (AOR = 1.072, 95% CI 1.020–1.127, *p *= 0.006), LVMDVi (AOR = 1.069, 95% CI 1.015–1.126, *p *= 0.012), and LVMSVi (AOR = 1.084, 95% CI 1.007–1.168, *p* = 0.032) were independently associated with CMD status in adjusted logistic regression models. These principal multivariable associations remained significant after correction for false discovery rate (all Q <0.05).

**Conclusions:**

CMD was associated with adverse left-heart remodeling, as manifested by myocardial hypertrophy, eccentric left ventricular remodeling, atrial dilation, and indices suggestive of early diastolic alterations.

## 1. Introduction

Coronary artery disease (CAD) is a chronic inflammatory atherosclerotic disorder and a major cause of cardiovascular morbidity and mortality worldwide [1]. Based on the severity of luminal narrowing, CAD can be classified as obstructive (lumen stenosis ≥50%) or non-obstructive (stenosis <50%) [2]. The diagnostic and therapeutic framework for obstructive CAD is well established because its ischemic mechanism, such as blood flow limitation, is clearly defined [2,3,4]. However, the clinical significance of non-obstructive CAD has historically been underestimated. Maddox et al. reported that patients with non-obstructive CAD had a 2–4.5-fold higher risk of myocardial infarction (MI) within one year compared to patients without overt CAD, with the risk increasing progressively according to the number of diseased vessels [5]. Therefore, the clinical risk associated with non-obstructive CAD has received increasing attention. Coronary microvascular dysfunction (CMD) is considered a key pathological mechanism underlying non-obstructive CAD [6,7,8,9]. CMD is defined as structural and functional impairment of the coronary microvasculature (arterioles and capillaries <400 μm) [7], leading to myocardial hypoperfusion. Ischemia with non-obstructive coronary artery disease (INOCA) accounts for a substantial proportion of non-obstructive CAD [10]. CMD is present in most patients with INOCA [11], and is strongly associated with increased risks of myocardial ischemia, heart failure with preserved ejection fraction (HFpEF), and adverse cardiovascular events, including MI, stroke, and heart failure (HF) [7,8,12,13].

The association between CMD and cardiac remodeling has attracted increasing attention. Previous studies have reported an association between coronary microvascular dysfunction and left ventricular (LV) diastolic dysfunction [14]. In addition, insufficiency of microcirculatory perfusion caused by CMD can induce subclinical myocardial ischemia, promote interstitial fibrosis, and increase ventricular stiffness, ultimately resulting in ventricular remodeling [15]. This pathological process has been reported in chronic heart failure (CHF), hypertrophic cardiomyopathy (HCM), hypertensive heart disease, and aortic stenosis [15,16,17]. Abnormal LV geometry is predictive of poor outcome in individuals without obstructive CAD [18]. However, in patients with INOCA, most studies to date have focused on the relationship between CMD and LV diastolic function, while the effects of CMD on LV systolic function and overall left-heart structure have received only limited attention. Therefore, characterizing the imaging features of early left-heart remodeling associated with CMD in patients with INOCA may improve disease phenotyping and provide insights into disease progression. This could also inform future investigations of potential therapeutic strategies. Most previous investigations of CMD-related cardiac remodeling have primarily relied on echocardiography and cardiac magnetic resonance (CMR) [14,19]. Coronary computed tomography angiography (CCTA) is a non-invasive imaging technology that has evolved beyond coronary lumen assessment, thanks to advances in scanner technology and post-processing algorithms. In addition to high-resolution visualization of coronary anatomy, CCTA enables quantitative evaluation of LV remodeling through assessments of left ventricular mass (LVM), left ventricular mid-diastolic volume (LVMDV), the concentricity index (CI; LVM/LVMDV), and left ventricular mid-diastolic wall thickness (LVMDWT) [20,21,22,23]. CCTA also enables feasible and reproducible volumetric assessment of left atrial (LA) remodeling. Recent studies have shown that CT-based LA volumetry is clinically applicable, with good agreement between measurements obtained at end-systole and mid-diastole [24,25,26]. Moreover, comparative studies have demonstrated that CT-derived LA size and function correlate well with CMR [27]. In addition to structural assessment, CCTA also enables the noninvasive evaluation of left-heart function. LV systolic function can be assessed by left ventricular ejection fraction (LVEF) [28], whereas diastolic function may be further characterized by the diastolic expansion (DE) index, defined as the ratio of LVMDV to left atrial mid-diastolic volume (LAMDV) [29]. Therefore, CCTA offers an integrated framework for the quantitative assessment of left-heart remodeling. By integrating multimodal CCTA parameters, we aimed to systematically characterize the imaging features of early left-heart remodeling (including structural and functional alterations) associated with CMD in patients with INOCA.

## 2. Methods

### 2.1 Study Population

This single-center cross-sectional study was conducted retrospectively. Consecutive patients presenting with chest pain, chest tightness, or other symptoms suggestive of myocardial ischemia between January 2020 and March 2024 were included. All patients underwent CCTA and invasive coronary physiological assessment within one week, including measurement of fractional flow reserve (FFR), coronary flow reserve (CFR), and index of microcirculatory resistance (IMR).

**Fig. 1.**
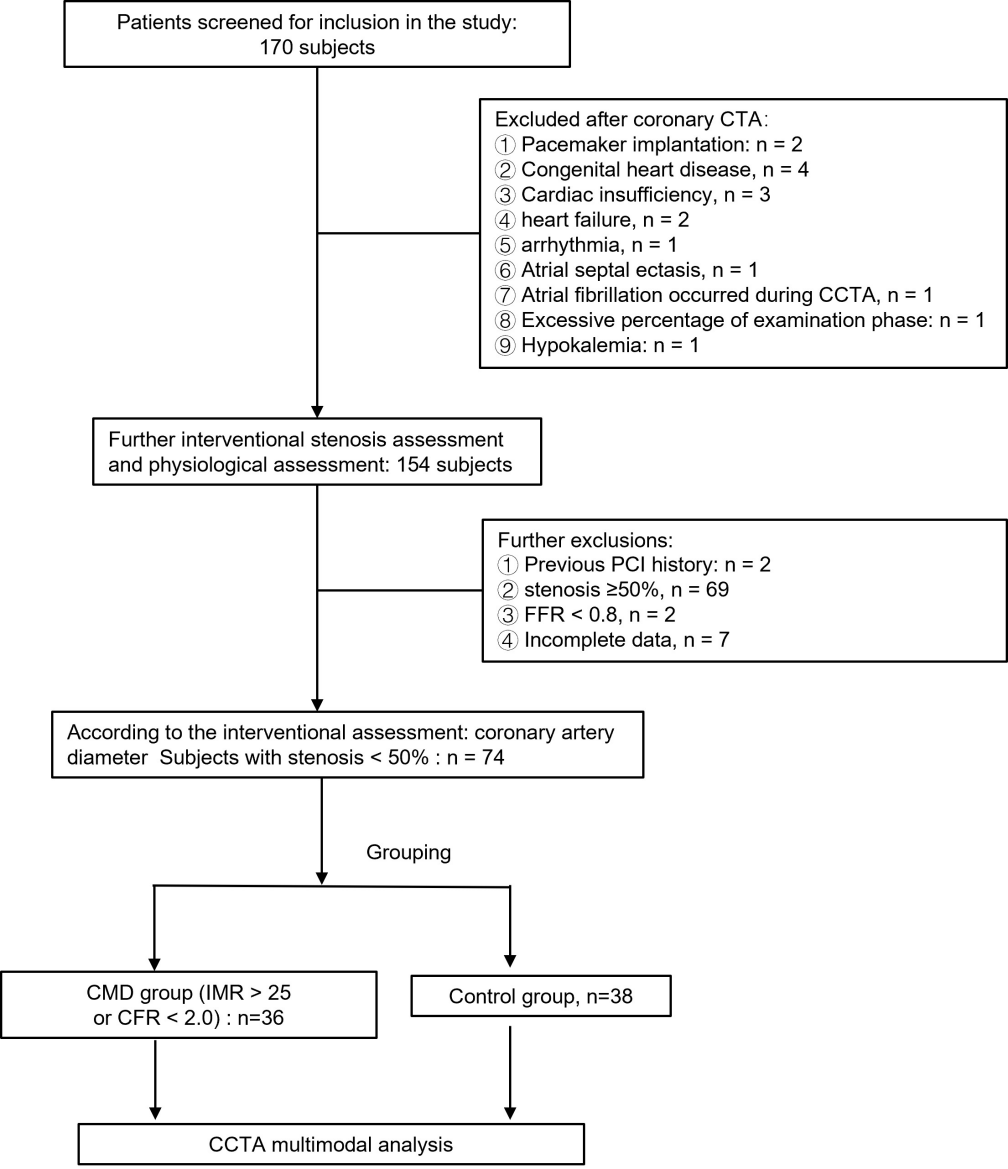
**Flow chart of patient screening. **CTA, computed tomography angiography; PCI, percutaneous coronary intervention; FFR, fractional flow reserve; CMD, coronary microvascular dysfunction; IMR, index of microcirculatory resistance; CCTA, coronary computed tomography angiography.

The exclusion criteria were as follows: (1) epicardial coronary stenosis ≥50%, or FFR <0.8; (2) history of cardiac surgery or MI; (3) severe structural heart disease (congenital heart disease, hypertrophic/dilated cardiomyopathy, severe valvular disease, etc.); (4) HF, cardiac dysfunction, or significant arrhythmia; (5) presence of other severe systemic disease; or (6) incomplete clinical data or poor image quality. A total of 74 patients were included in the final analysis, with 36 patients in the CMD group (IMR >25 or CFR< 2.0) and 38 patients in the control group (Fig. 1).

### 2.2 CCTA Examination Protocol

A Siemens SOMATOM Definition Flash CT scanner (Siemens AG, Forchheim, Germany; serial number: 73486) was used for imaging. The parameters were set according to the Chinese Expert Consensus on Examination and Diagnosis of CT for Coronary Atherosclerotic Heart Disease (2024) [30], with automatic tube current modulation (CARE Dose4D), 100 kVp tube voltage (optimized for body mass index (BMI) <25 kg/m^2^), 280 ms gantry rotation time, and 128 × 0.6 mm collimation. Prospective ECG-gated acquisition was performed during mid-diastole (70% of the R-R interval) with a 75 ms acquisition window. A dual-syringe and two-phase injection protocol was used. Iodinated contrast agent (Iodixanol 370) followed by normal saline was injected at a rate of 5.0 mL/s. All patients received oral β-blockers to maintain a heart rate of <65 beats/min. Intracoronary nitroglycerin (0.8 mg, Guangzhou Baiyun Mingxing Pharmaceutical Co., Ltd., Guangzhou, Guangdong, China) was administered to optimize coronary artery visualization. The B26f medium-smooth ASA algorithm reconstruction was used for iterative reconstruction, with a slice thickness of 0.75 mm and a slice interval of 0.5 mm. Images were reconstructed in at least three cardiac phases, including the optimal diastolic phase and the boundaries of the acquisition window, and subsequently transferred to dedicated post-processing software for further analysis.

### 2.3 Assessment of Coronary Artery Stenosis

Coronary artery stenosis was evaluated using invasive coronary angiography (CAG). High-quality images of the four major epicardial coronary arteries were obtained in at least two orthogonal projections (angular difference ≥25º) to reduce artifacts caused by vessel foreshortening or overlap. These were the left main coronary artery (LMCA), left anterior descending artery (LAD), left circumflex artery (LCX), and right coronary artery (RCA). Quantitative coronary angiography (QCA) was performed using AngioPlus software (Pulse Medical Imaging Technology, Shanghai, China). This was conducted offline by two independent, experienced technicians who were blinded to clinical information. A validated and automated edge-detection algorithm was used to determine the reference vessel diameter (measured proximal and distal to the lesion) and the minimal luminal diameter at the most stenotic segment. Patients with stenosis ≥50% as identified by QCA were excluded from the study.

### 2.4 IMR and CFR Assessment Protocol

Coronary microvascular function was invasively evaluated using a pressure- and temperature-sensor-tipped guidewire (PressureWire X, St. Jude Medical, USA) based on the intracoronary thermodilution method. This was performed according to standardized procedures for the measurement of IMR and CFR.

Prior to intracoronary deployment, the guidewire underwent a multi-step calibration process. (1) Following air removal with heparinized saline, the guidewire was connected to the acquisition system. Zero calibration of the aortic pressure (Pa) and guidewire pressure was performed *in situ* with the wire positioned horizontally at the level of the mid-axillary line. (2) The sensor tip was advanced to the coronary ostium. After flushing the guiding catheter with saline to remove residual contrast, the Y-valve was closed and pressure equalization (EQ) was performed to eliminate the pressure gradient between proximal Pa and distal coronary pressure (Pd), ensuring a Pd/Pa ratio of 1.0. Stability was verified by recording five consecutive cardiac cycles. (3) Temperature sensor calibration was performed using three consecutive intracoronary bolus injections (3 mL of 0.9% saline at room temperature) delivered within ≤0.6 s. Each injection was considered valid when the fall in temperature exceeded 2 ºC. Only datasets with three consecutive valid measurements were accepted.

Baseline stability was confirmed before data acquisition as follows: (1) intracoronary nitroglycerin (200 μg) was administered to prevent epicardial vasospasm; (2) hemodynamic stability was verified, with mean arterial pressure returning to a stable baseline without significant fluctuations or arrhythmias; (3) optimal sensor positioning was ensured, with the pressure-temperature guidewire advanced 2–3 cm distal to the target lesion (or beyond two-thirds of the vessel length in highly tortuous segments), while maintaining a fixed alignment between the guiding catheter and guidewire to avoid motion artifacts.

Data recording (“REC”) was initiated at rest. Resting parameters were obtained from three consecutive bolus injections of 3 mL saline at room temperature, each delivered within 0.6 s (measurements were repeated if any value deviated by >30% from the mean of the other two values). Maximal hyperemia was then induced by continuous intravenous infusion of adenosine 5′-triphosphate (ATP) at 140 μg/kg/min. Adequate hyperemia was confirmed when Pa decreased by 10–20%, and the Pa, Pd, and Pd/Pa curves became stable and parallel for at least 20 consecutive seconds. Under stable maximal hyperemia, three additional saline boluses were administered to determine the hyperemic mean transit time (Tmn). Measurements were repeated if any value deviated by more than 30% from the mean of the other two values. Data recording and ATP infusion were stopped after completion of the measurements. The final FFR and CFR values were displayed automatically, whereas IMR was calculated manually (IMR = Pd × Tmn).

### 2.5 Post-Processing and Analysis of Images

**Fig. 2.**
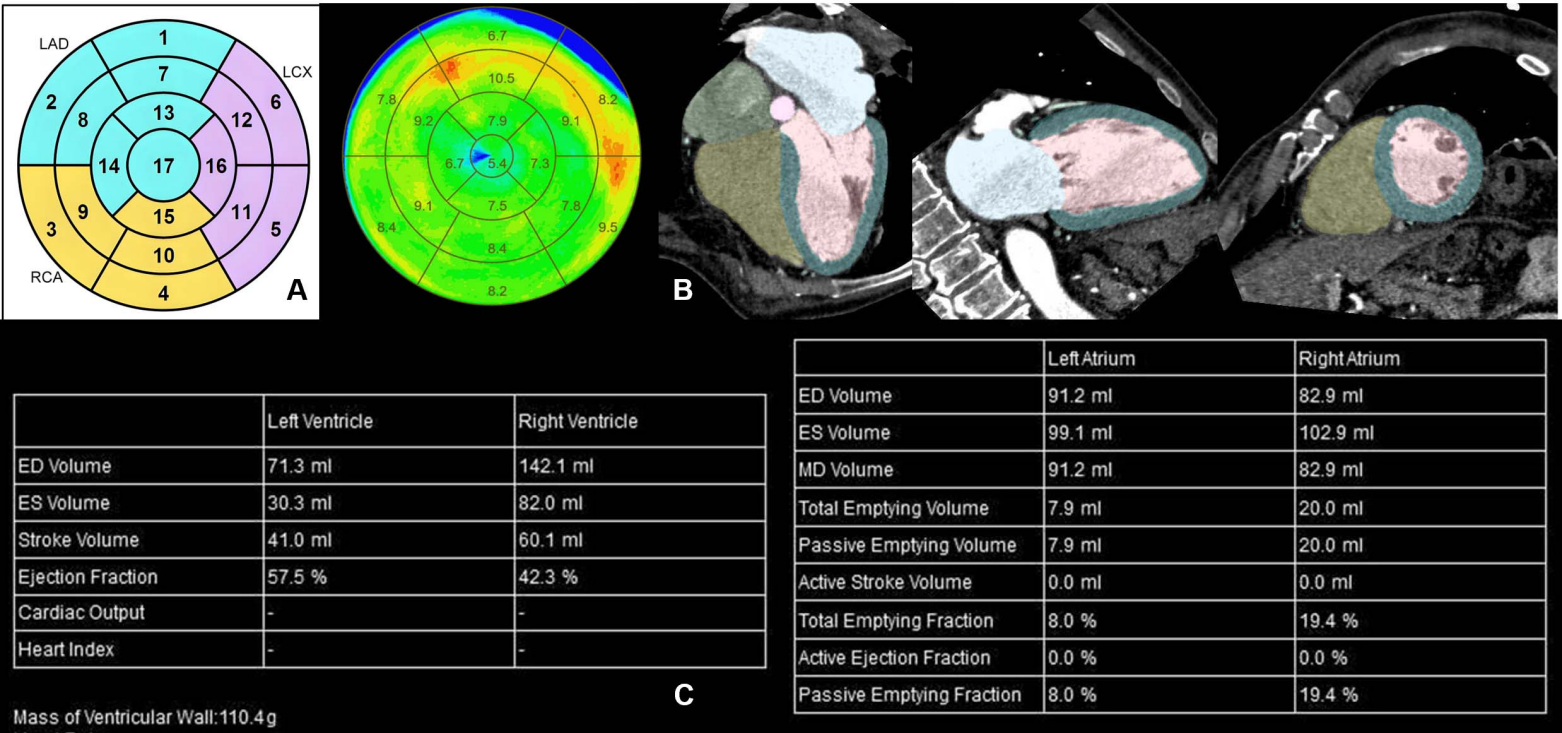
**Representative images and derived CCTA parameters**. (A) The 17-segment model and LV wall thickness measurements. (B) Four-chamber, short-axis, and two-chamber views showing automatic segmentation of endocardial and epicardial contours. (C) CCTA-derived parameters obtained after correction on the UNITED IMAGING post-processing workstation (LVM, LVMDV, LVMSV, LAMDV, LAMSV, LVEF). LAD, left anterior descending artery; LCX, left circumflex artery; RCA, right coronary artery; ED, end-diastolic; ES, end-systolic; MD, mid-diastolic; CCTA, coronary computed tomography angiography; LVM, left ventricular mass; LVMDV, left ventricular mid-diastolic volume; LVMSV, left ventricular mid-systolic volume; LAMDV, left atrial mid-diastolic volume; LAMSV, left atrial mid-systolic volume; LVEF, left ventricular ejection fraction.

The UNITED IMAGING post-processing workstation uWS-CT was used to automatically segment the epicardial and endocardial borders, with manual adjustment of contouring when necessary, based on the phases with maximal and minimal LV volume (Fig. 2). After correction, papillary muscles were excluded from LVM calculation and included in the blood-pool volume measurement.

The LVMDV, LAMDV, and LVM were measured at the phase with the maximal LV volume, whereas the left ventricular mid-systolic volume (LVMSV) and left atrial mid-systolic volume (LAMSV) were obtained at the phase with minimal LV volume (Fig. 2). LVM, LVMDV, LVMSV, LAMDV, and LAMSV were indexed to body surface area (BSA) using the Mosteller formula, yielding LVMi, LVMDVi, LVMSVi, LAMDVi, and LAMSVi, respectively. To evaluate LV geometric remodeling, the calculation methods used in echocardiography and CMR were adapted to mid-diastolic LV measurements, and the modified CCTA CI was defined as LVM/LVMDV. Using the 17-segment model, the system automatically measures LV wall thickness for each corresponding segment (Fig. 2). Apical segments were often unreliable and were therefore excluded [22]. LVMDWT was calculated as the mean thickness of the remaining 16 LV segments. LV systolic function was assessed using LVEF, derived from mid-diastolic and mid-systolic volumes. Diastolic function was evaluated using LAMDV and LAMSV indexed to BSA, as well as the DE, defined as LVMDV/LAMDV.

### 2.6 Statistical Analysis

Normality was evaluated using the Shapiro-Wilk test. Continuous variables were expressed as the mean ± standard deviation (SD) for normally distributed data, or as the median (interquartile range) for non-normally distributed data. Categorical variables were presented as counts and percentages. Between-group comparisons were performed using the independent-samples *t*-test for normally distributed variables, the Mann-Whitney U test for non-normally distributed variables, and the chi-square test (or Fisher’s exact test when appropriate) for categorical variables. CMD status was used as the dependent variable in binary logistic regression models. Because of the limited number of events and potential intercorrelations among CT parameters, each CT remodeling parameter was entered separately into the multivariable model to minimize overfitting. The primary multivariable model was adjusted for age, sex, and hypertension. Multicollinearity among predictors was assessed using variance inflation factors (VIFs) (**Supplementary 1-Supplementary Methods**).

To account for multiple testing in both the multiple comparisons among CCTA-derived remodeling parameters and the primary multivariable logistic regression models, the false discovery rate (FDR) was controlled using the Benjamini-Hochberg procedure, and FDR-adjusted *p*-values (Q-values) were reported. Additional sensitivity analyses were conducted, including further adjustment for BMI (**Supplementary Table 1**) and alternative body-size adjustment using non-indexed (raw) CT parameters with BSA as a covariate. Results were reported as odds ratios (ORs) with 95% confidence intervals (CIs) (**Supplementary Table 3**). To examine the robustness of the findings to alternative CFR thresholds (i.e., sensitivity analysis), the main analyses, including multivariable logistic regression, were repeated using an alternative CMD definition (IMR >25 or CFR <2.5). Detailed results are presented in **Supplementary Tables 4,5**. All analyses were performed using IBM SPSS Statistics, Version 26.0 (IBM Corp., Armonk, NY, USA), with a *p*-value < 0.05 considered to indicate statistical significance.

## 3. Results

### 3.1 Baseline Demographics

Among the 74 patients, 36 were assigned to the CMD group (IMR >25 or CFR <2.0) and 38 to the control group. No significant differences in baseline characteristics were observed between the two groups (*p *> 0.05; Table 1).

**Table 1.** **Baseline characteristics**.

Variable	Total	Non-CMD	CMD	Statistic	*p*
	n = 74	n = 38	n = 36		
Age (years)	62.22 ± 9.61	61.82 ± 10.39	62.64 ± 8.84	t = –0.37	0.715
Sex (male, %)	34 (45.946)	18 (47.368)	16 (44.444)	χ^2^ = 0.064	0.801
BMI (kg/m^2^)	25.28 ± 3.13	25.28 ± 3.34	25.29 ± 2.93	t = –0.01	0.993
BSA	1.74 ± 0.16	1.76 ± 0.15	1.72 ± 0.16	t = 0.87	0.389
SBP (mmHg)	136.97 ± 21.49	137.29 ± 23.38	136.63 ± 19.57	t = 0.13	0.897
DBP (mmHg)	79.00 ± 13.95	78.05 ± 13.99	80.03 ± 14.03	t = –0.60	0.549
Pulse	76.62 ± 12.35	78.32 ± 11.72	74.77 ± 12.91	t = 1.23	0.223
Hypertension (%)	43 (58.108)	23 (60.526)	20 (55.556)	χ^2^ = 0.188	0.665
Diabetes (%)	15 (20.270)	6 (15.789)	9 (25.000)	χ^2^ = 0.970	0.325
Dyslipidemia (%)	20 (27.027)	11 (28.947)	9 (25.000)	χ^2^ = 0.146	0.702
Smoking (%)	14 (18.919)	6 (15.789)	8 (22.222)	χ^2^ = 0.499	0.480
Urea (mmol/L)	5.67 ± 1.60	5.72 ± 1.73	5.64 ± 1.54	t = 0.16	0.872
CR (μmol/L)	81.63 ± 15.10	81.55 ± 16.19	81.68 ± 14.62	t = –0.03	0.977
UA (μmol/L)	354.51 ± 91.95	382.70 ± 92.29	336.32 ± 88.46	t = 1.80	0.078
FBG (mmol/L)	5.23 ± 1.05	5.33 ± 1.31	5.16 ± 0.86	t = 0.58	0.564
TG (mmol/L)	1.85 ± 1.23	2.15 ± 1.40	1.56 ± 0.97	t = 1.97	0.053
TC (mmol/L)	4.35 ± 1.01	4.49 ± 1.06	4.22 ± 0.95	t = 1.09	0.281
HDL-C (mmol/L)	1.14 ± 0.28	1.14 ± 0.26	1.14 ± 0.29	t = –0.08	0.936
LDL-C (mmol/L)	2.45 ± 0.81	2.60 ± 0.85	2.32 ± 0.77	t = 1.37	0.099

Abbreviations: CMD, coronary microvascular dysfunction; BMI, body mass index; CR, creatinine; UA, uric acid; FBG, fasting blood glucose; TG, triglycerides; TC, total cholesterol; HDL-C, high-density lipoprotein cholesterol; LDL-C, low-density lipoprotein cholesterol; BSA, body surface area; SBP, systolic blood pressure; DBP, diastolic blood pressure.

### 3.2 Imaging Features

#### 3.2.1 Structural Parameters

**Fig. 3.**
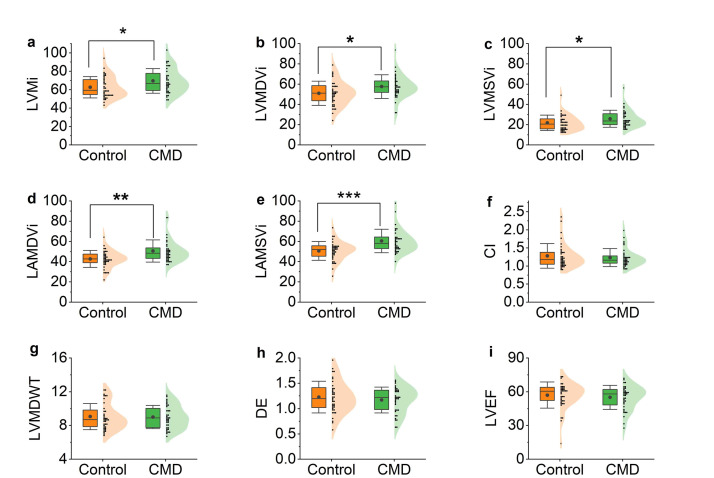
**Half-violin and boxplot comparison of left-heart remodeling parameters between the control group (orange) and CMD group (green)**. (a–g**)** Represent structural parameters. (d,e,h,i) represent functional parameters. Abbreviations: CMD, coronary microvascular dysfunction; i, indexed to body surface area; LVMi, left ventricular mass index; LVMDVi, left ventricular mid-diastolic volume index; LVMSVi, left ventricular mid-systolic volume index; LAMDVi, left atrial mid-diastolic volume index; LAMSVi, left atrial mid-systolic volume index; CI, concentricity index (LVM/LVMDV); LVMDWT, left ventricular mid-diastolic wall thickness; DE, diastolic expansion index (LVMDV/LAMDV); LVEF, left ventricular ejection fraction; *p < 0.05; **p < 0.01, ***p < 0.001.

No significant differences were found between the CMD and control groups in terms of LVM (*p* = 0.113) and LVMDV (*p* = 0.080), although significant differences were observed after indexing for BSA (*p *= 0.023 and* p *= 0.018, respectively). LVMSV was significantly higher in the CMD group than in the control group (*p *= 0.025), with this difference remaining significant after indexing for BSA (*p *= 0.017). LAMDV (*p *= 0.003) and LAMSV (*p *< 0.001) were also significantly higher in the CMD group, and again these differences remained after indexing for BSA (*p *= 0.002 and *p* < 0.001, respectively). All significant differences remained after adjustment for multiple comparisons using the FDR method (all Q values <0.05). However, no significant differences between the CMD and control groups were observed for CI (*p *= 0.868) or LVMDWT (*p *= 0.879) (Table 2, Fig. 3a–g).

**Table 2.** **Parameters of left-heart remodeling**.

Variable	Total	Non-CMD	CMD	Statistic	*p*	Q-Value	95% CI	Effect Size
	n = 74	n = 38	n = 36					
LVM (g)	111.00 (94.10, 133.85)	103.80 (91.80, 131.55)	113.55 (99.38, 138.90)	Z = –1.58	0.113	0.158	–21.100, 2.500	0.364
LVMi (g/m^2^)	63.98 (55.09, 74.40)	59.43 (54.66, 70.68)	66.68 (59.36, 76.63)	Z = –2.28	0.023*	0.044*	–12.090, –1.156	0.559
LVMDV (mL)	94.41 ± 23.32	89.79 ± 24.26	99.28 ± 21.56	t = –1.77	0.080	0.124	–20.147, 1.179	0.413
LVMDVi (mL/m^2^)	54.20 ± 12.19	50.95 ± 11.91	57.62 ± 11.67	t = –2.43	0.018*	0.042*	–12.137, –1.199	0.565
LVMSV (mL)	37.70 (30.83, 47.40)	34.60 (27.52, 46.83)	38.95 (36.12, 51.90)	Z = –2.24	0.025*	0.044*	–11.400, –0.700	0.434
LVMSVi (mL/m^2^)	22.41 (17.88, 28.27)	20.63 (15.86, 25.65)	23.57 (19.99, 30.22)	Z = –2.38	0.017*	0.042*	–6.891, –0.746	0.515
LAMDV (mL)	80.55 ± 17.74	74.68 ± 15.35	86.75 ± 18.17	t = –3.09	0.003*	0.011*	–19.853, –4.294	0.720
LAMDVi (mL/m^2^)	45.21 (41.08, 50.60)	42.70 (39.05, 47.21)	48.40 (43.32, 53.52)	Z = –3.17	0.002*	0.009*	–10.107, –2.346	0.808
LAMSV (mL)	96.03 ± 19.77	88.74 ± 17.38	103.73 ± 19.42	t = –3.50	<0.001*	0.007*	–23.523, –6.459	0.815
LAMSVi (mL/m^2^)	55.40 ± 11.55	50.64 ± 9.35	60.42 ± 11.62	t = –4.00	<0.001*	0.007*	–14.657, –4.903	0.930
CI (g/mL)	1.17 (1.07, 1.35)	1.18 (1.06, 1.38)	1.16 (1.08, 1.27)	Z = –0.17	0.868	0.935	–0.083, 0.110	0.154
LVMDWT (mm)	9.03 ± 1.46	9.05 ± 1.56	9.00 ± 1.37	t = 0.15	0.879	0.879	–0.628, 0.733	0.036
DE	1.20 ± 0.29	1.23 ± 0.31	1.17 ± 0.25	t = 0.84	0.406	0.474	–0.770, 0.188	0.194
LVEF (%)	58.38 (51.42, 63.19)	60.49 (52.24, 63.82)	58.04 (48.70, 61.54)	Z = –1.06	0.289	0.368	–2.177, 6.763	0.179

Abbreviations: CMD, coronary microvascular dysfunction; i, indexed to body surface area; LVM, left ventricular mass; LVMi, left ventricular mass index; LVMDV, left ventricular mid-diastolic volume; LVMDVi, left ventricular mid-diastolic volume index; LVMSV, left ventricular mid-systolic volume; LVMSVi, left ventricular mid-systolic volume index; LAMDV, left atrial mid-diastolic volume, LAMDVi: left atrial mid-diastolic volume index; LAMSV, left atrial mid-systolic volume; LAMSVi, left atrial mid-systolic volume index; CI, concentricity index (LVM/LVMDV); LVMDWT, left ventricular mid-diastolic wall thickness; DE, diastolic expansion index (LVMDV/LAMDV); LVEF, left ventricular ejection fraction; *p < 0.05. Q-value, Benjamini-Hochberg-adjusted *p*-value; *Q <0.05 (Q-values were calculated across all 14 remodeling parameters).

#### 3.2.2 Functional Parameters

LAMDV (*p *= 0.003) and LAMSV (*p* < 0.001) were significantly higher in the CMD group than in the control group, with the differences persisting after indexing for BSA (*p *= 0.002 and *p* < 0.001, respectively). In contrast, no significant differences between the two groups were detected for DE (*p *= 0.406) or LVEF (*p *= 0.289) (Table 2, Fig. 3d,e,h,i).

#### 3.2.3 Multivariable Logistic Regression Analysis

In multivariable logistic regression models adjusted for age, sex, and hypertension, higher LAMSVi, LAMDVi, LVMi, LVMDVi, and LVMSVi were independently associated with CMD (*p* < 0.001, *p* = 0.002, *p* = 0.006, *p* = 0.012, and *p* = 0.032, respectively) (Table 3). Importantly, these associations remained statistically significant after FDR correction across the five primary models (Q = 0.004, Q = 0.004, Q = 0.010, Q = 0.015, and Q = 0.032, respectively). Sensitivity analyses with additional adjustment for BMI produced similar results (**Supplementary Table 1**). Analyses based on raw CCTA parameters, with additional adjustment for BSA, also yielded consistent associations, supporting the robustness of the findings across alternative body-size adjustment methods (**Supplementary Tables 2,3**).

**Table 3.** **Univariate and multivariate logistic regression analysis of CMD**.

Variable	Univariate		Multivariate			Q-value	
	OR (95% CI)	*p*	OR (95% CI)		*p*		
LAMSVi	1.114 (1.043–1.190)	0.001	1.129 (1.052–1.211)	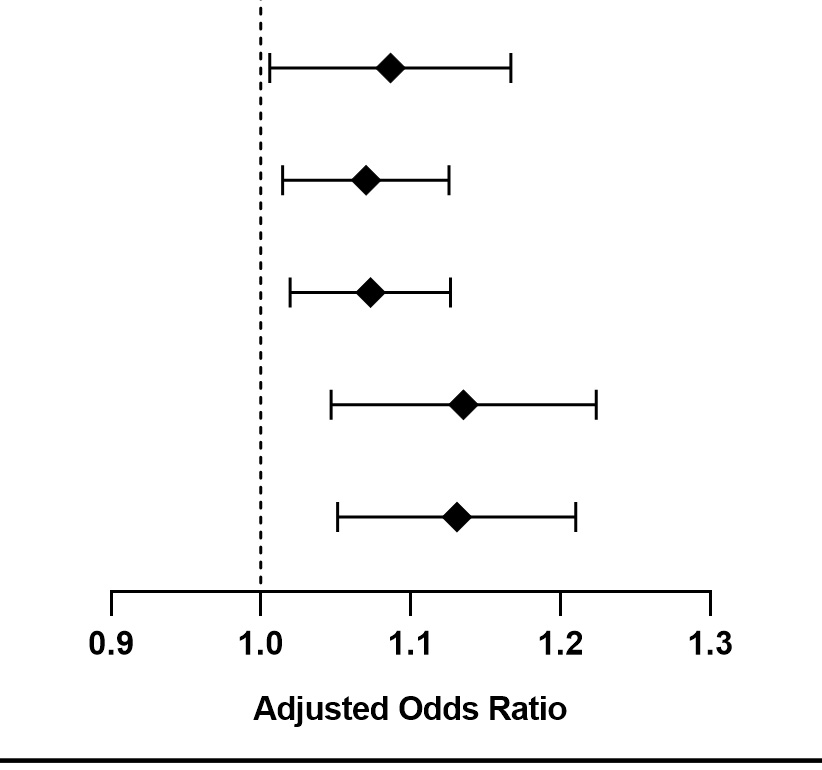	0.001*	0.004	
LAMDVi	1.106 (1.033–1.184)	0.004	1.133 (1.048–1.225)		0.002	0.004	
LVMi	1.047 (1.006–1.089)	0.024	1.072 (1.020–1.127)		0.006	0.010	
LVMDVi	1.052 (1.007–1.098)	0.024	1.069 (1.015–1.126)		0.012	0.015	
LVMSVi	1.072 (1.004–1.146)	0.039	1.084 (1.007–1.168)		0.032	0.032	

The forest plot presents adjusted odds ratios (ORs) with 95% confidence intervals (CIs). Left panel: results from univariate analysis. Right panel: results from multivariate analysis adjusted for age, sex, and hypertension. Note: To minimize multicollinearity and reduce the risk of overfitting (based on the EPV principle), each CT parameter was entered separately into the multivariable logistic regression model together with the covariates. Q-value, Benjamini-Hochberg-adjusted *p*-value; *Q <0.05 (Q-values were calculated across 5 remodeling parameters). Abbreviations: LAMSVi, left atrial mid-systolic volume index; LAMDVi, left atrial mid-diastolic volume index; LVMi, left ventricular mass index; LVMDVi, left ventricular mid-diastolic volume index; LVMSVi, left ventricular mid-systolic volume index.

### 3.3 Coronary Physiological Characteristics

Epicardial physiological indices were comparable between the CMD and control groups, with no significant differences in FFR, Pa, or Pd (Table 4). In contrast, the CMD group exhibited clear microvascular dysfunction, characterized by significantly higher IMR, longer Tmn, and lower CFR.

**Table 4.** **Coronary physiological characteristics**.

Variable		Total	Non-CMD	CMD	Statistic	*p*
		n = 74	n = 38	n = 36		
Epicardial indices						
	FFR	0.91 ± 0.06	0.92 ± 0.05	0.91 ± 0.06	t = 0.76	0.451
	Pa (mmHg)	78.50 (69.25, 87.75)	79.50 (71.50, 88.25)	77.50 (69.00, 87.25)	Z = –0.23	0.816
	Pd (mmHg)	72.00 (62.25, 82.00)	74.00 (63.25, 82.00)	70.50 (62.75, 81.25)	Z = –0.45	0.649
	Tmn (s)	0.36 ± 0.22	0.22 ± 0.08	0.51 ± 0.22	t = –7.59	<0.001*
Microvascular indices						
	IMR	26.43 ± 16.22	15.80 ± 4.88	37.66 ± 16.47	t = –7.65	<0.001*
	CFR	3.80 (2.55, 5.42)	4.40 (3.73, 6.55)	2.95 (1.65, 4.30)	Z = –4.07	<0.001*

Abbreviations: CMD, coronary microvascular dysfunction; FFR, fractional flow reserve; Pa, proximal aortic pressure; Pd, distal coronary pressure; Tmn, mean transit time; IMR, index of microcirculatory resistance; CFR, coronary flow reserve; *p < 0.05. CMD was defined as IMR >25 or CFR <2.0, and consequently, CFR may remain preserved in a subset of CMD patients with elevated IMR.

### 3.4 Sensitivity Analysis Using an Alternative CFR Cutoff

In sensitivity analyses using an alternative CFR threshold (IMR >25 or CFR <2.5), two additional patients were reclassified as CMD (CMD, n = 38; non-CMD, n = 36). Group comparisons showed that LAMSVi, LAMDVi, and LVMi remained significantly different between the two groups, whereas differences in LVMDVi and LVMSVi were reduced and no longer statistically significant (**Supplementary Table 4**). Similarly, in sensitivity multivariable logistic regression analyses based on this alternative CFR threshold, higher LAMSVi, LAMDVi, and LVMi remained independently associated with CMD (*p* = 0.002, *p* = 0.002, and *p* = 0.010, respectively). However, the associations for LV volumetric indices were attenuated, with LVMDVi showing only borderline significance (*p* = 0.049) and LVMSVi becoming non-significant (*p* = 0.063) (**Supplementary Table 5**).

## 4. Discussion

Our study found that CMD in INOCA patients was independently associated with eccentric left ventricular hypertrophy and left atrial dilation, as quantified by various CCTA-derived indices, including LVMi, left ventricular volume index (LVVi, including LVMDVi and LVMSVi), and left atrial volume index (LAVi, including LAMDVi and LAMSVi). These structural alterations, particularly left atrial enlargement, may indicate early diastolic-related changes. However, because DE did not differ significantly between the CMD and control groups, and conventional echocardiographic or CMR diastolic indices were unavailable, this interpretation should be considered exploratory and hypothesis-generating. To our knowledge, this is the first study to integrate cardiac structural and functional parameters using CCTA, providing new evidence for the early imaging assessment of CMD. The consistency, discrepancies, and theoretical implications of these findings are discussed in the context of existing literature.

At the structural level, the present study showed that CMD is associated with significant left-heart structural remodeling, characterized by increased LVMi, LVVi, and LAVi. This finding is consistent with several previous studies [14,31]. However, no significant differences were observed in LVMDWT or CI. Mechanistically, CMD-induced microvascular ischemia may promote cardiomyocyte apoptosis and necrosis, thereby activating fibroblasts and increasing collagen deposition. This process leads to myocardial interstitial fibrosis and increased myocardial mass [8]. At the same time, increased myocardial stiffness and wall stress may result in compensatory ventricular dilation [14]. Importantly, our results suggest that myocardial mass and ventricular volume increase simultaneously. This parallel increase maintains a relatively stable mass-to-volume ratio, which may explain the lack of association with the CI. This pattern is also consistent with eccentric remodeling rather than concentric hypertrophy [16,32,33]. In addition, the left atrium may preserve ventricular filling by increasing contractility and enlarging its volume. With progression of CMD, the atrium may be exposed to chronic pressure overload, eventually leading to structural remodeling such as atrial dilation and fibrosis.

In our cohort, CMD status was independently associated with CCTA-derived functional remodeling features, particularly an increase in LAVi. Because LAVi is widely associated with diastolic dysfunction and increases with its severity [34,35], the higher LAVi observed in CMD patients may be consistent with early diastolic alterations. However, conventional echocardiographic or CMR diastolic indices were unavailable, and the study design was cross-sectional, this interpretation should be considered exploratory and hypothesis-generating. The current findings are consistent with previous reports linking microvascular dysfunction to diastolic abnormalities. Although this supports the plausibility of our observations, further prospective studies are required to clarify the temporal relationships. The literature suggests that DE may serve as a sensitive indicator of left ventricular filling pressure and diastolic dysfunction, and a reduced DE is associated with increased LA pressure and impaired LV relaxation [29,36,37]. However, in our cohort, DE did not differ significantly between the CMD and control groups, and therefore a definitive conclusion regarding DE and diastolic dysfunction cannot be drawn from this work. Rather, our findings suggest that CCTA-derived volumetric coupling indices are less sensitive to subtle early functional abnormalities than atrial volumetric remodeling. In contrast to an earlier imaging study [14], we did not observe a significant association between CMD and LVEF. Although our data do not permit definitive functional classification, preserved LVEF in the presence of structural remodeling may be compatible with an early HFpEF-like stage in which global systolic function remains preserved. The enlarged LA volume observed here may reflect the burden of chronic remodeling, or possibly an early filling abnormality, but it cannot independently establish diastolic dysfunction. Accordingly, LV mass, LV volume, and LA volume may be more sensitive markers of CMD-related remodeling than LVEF. CMD-related ischemia is often patchy rather than transmural, which may allow global systolic function to remain preserved despite regional microvascular impairment. To our knowledge, the present study is the first to comprehensively evaluate CMD-related left-heart remodeling in patients with INOCA using CCTA combined with invasive physiological assessment, thereby addressing a technical gap in the application of CCTA for CMD evaluation. The high spatial resolution of CCTA provides detailed information on cardiac chamber size and global LVM, which shows strong agreement with CMR [23,38]. Semi-automated image analysis may also help to reduce operator-related measurement variability. Evidence that LV mid-diastolic measurements correlate closely with LV end-diastolic measurements supports the validity of CCTA-derived indices, and both measurements have prognostic significance for major adverse cardiac events (MACE) [39,40,41]. To our knowledge, this is the first study to demonstrate that CCTA-derived LAVi is an independent predictor of CMD in INOCA patients, highlighting its potential as a non-invasive biomarker for early risk stratification and monitoring of microvascular dysfunction. Elevated LAVi may reflect microcirculatory ischemia caused by CMD, which increases atrial passive volume and restricts ventricular filling through myocardial fibrosis. This mechanism is consistent with the concept of atrioventricular coupling imbalance in the HFpEF model [42,43]. Therefore, LAVi may represent an early structural marker of CMD-related remodeling. As part of an evolving multimodal diagnostic framework for CMD, CCTA may provide complementary value beyond the exclusion of obstructive CAD by identifying structural phenotypes such as elevated LAVi and eccentric remodeling. These observations suggest that CCTA-derived markers may help to identify patients with INOCA who require further functional or invasive physiological evaluation, although prospective validation is necessary before routine clinical application.

Importantly, the clinical evaluation of CMD is shifting from a purely invasive strategy toward a comprehensive, patient-centered diagnostic approach. As recently reviewed by Polimeni et al. [44], non-invasive modalities such as stress echocardiography, CMR, and positron emission tomography (PET) play key roles in the initial screening and functional assessment of microvascular ischemia. Although PET remains the reference standard for quantifying myocardial blood flow and CMR provides superior tissue characterization, our findings suggest that CCTA may offer additional value beyond excluding obstructive CAD. By identifying structural phenotypes such as increased LAVi and eccentric remodeling, CCTA may integrate into this multimodal diagnostic pathway and potentially serve as a gatekeeper to identify patients who might benefit from further invasive physiological testing (e.g., IMR or CFR), as proposed in contemporary diagnostic workflows. The practicality and reproducibility of CCTA-based imaging, which requires no additional scanning or contrast administration, make it a useful complementary modality to echocardiography or CMR, particularly in patients who cannot tolerate other imaging examinations. However, larger studies are required to determine the optimal diagnostic thresholds and to clarify the dynamic relationship between CCTA-derived indices and hemodynamic parameters such as CFR and IMR. When combined with additional CCTA-derived functional indices (e.g., myocardial strain), increases in LVMi, LVVi, and LAVi may indicate the occurrence of early cardiac remodeling in patients with CMD before the onset of clinical symptoms. Identification of high-risk individuals based on imaging phenotypes may therefore provide an opportunity for earlier intervention.

Although the present cohort was modest in size, this is to be expected in studies involving the physiological characterization of INOCA/angina with non-obstructive coronary arteries (ANOCA) populations. Comprehensive coronary functional assessment is usually restricted to selected patients, and prior studies of comparable populations have reported similarly modest sample sizes. For example, Gao et al. studied 66 patients with INOCA using a physiology-based index of microvascular resistance. Therefore, although sample size remains a limitation, the scale of the present cohort is broadly in line with previous work in this field [45].

## 5. Limitations

First, because this study used a retrospective cross-sectional design, causal relationships cannot be established. In addition, conventional echocardiographic and CMR diastolic reference indices, including the E/A ratio, e' velocity, E/e' ratio, left atrial strain, and estimates of filling pressure, were not systematically available in this cohort. Therefore, CCTA-derived indices of LA volume, LV volume, and LVM should be interpreted primarily as markers of structural and volumetric remodeling rather than as direct functional surrogates. In particular, although increased CT-derived LAVi may reflect a chronically elevated filling burden, its functional interpretation in this study remains exploratory and hypothesis-generating, rather than definitive evidence of diastolic dysfunction. Similarly, because DE is derived from the simultaneous measurement of atrial and ventricular volumes, its value may also be influenced by technical differences among CCTA post-processing software platforms. To better clarify the mechanisms underlying these interactions and the dynamic progression of cardiac remodeling, future studies should incorporate longitudinal follow-up designs.

Second, this single-center study had a relatively small sample size (n = 74). As mentioned above, this limitation reflects the strict clinical indications required for invasive wire-based physiological testing (IMR and CFR) and the rigorous exclusion criteria applied to ensure a relatively homogeneous INOCA cohort without major confounders, such as significant epicardial stenosis or prior revascularization. Importantly, cohorts requiring invasive coronary function testing are typically limited in size. Despite this limitation, the reported effect sizes with 95% confidence intervals and FDR correction enhance the robustness of the observed associations. Nevertheless, the single-center design and limited sample size may introduce selection bias and restrict generalizability, and larger multicenter prospective studies across different disease stages are needed to validate our findings. Third, although CCTA is a widely accepted and non-invasive imaging modality with high diagnostic accuracy, its ability to evaluate subtle structural and functional parameters, such as myocardial perfusion, remains limited. Integration with other imaging modalities or multimodal data fusion may therefore improve diagnostic accuracy.

Fourth, CMD represents a heterogeneous spectrum that includes both impaired microcirculatory conductance and microvascular spasm. Although a full physiology framework may also include the resistive reserve ratio (RRR), this was not systematically acquired in our retrospective cohort. CMD classification in the present study was based on invasive IMR and CFR, while the vasospastic endotype was not evaluated. Definitive diagnosis of microvascular spasm requires acetylcholine provocation testing (AChT). However, intracoronary AChT is not routinely performed in our clinical practice because of safety considerations, particularly the risk of severe arrhythmias, as well as differences in clinical protocols within Asian populations [46,47]. Consequently, the potential influence of vasospastic endotypes on remodeling patterns in the present cohort cannot be completely excluded. In addition, the CMD group was defined using an “OR” criterion (IMR >25 or CFR <2.0), consistent with current clinical consensus for the diagnosis of INOCA [10]. However, this approach may introduce physiological heterogeneity, since IMR primarily reflects microcirculatory structural resistance, whereas CFR represents an integrated measure of global coronary flow capacity. Combining these distinct physiological dimensions into a single binary category may partially dilute effect sizes and attenuate associations for certain indices, as suggested by the sensitivity analysis using an alternative CFR threshold. This heterogeneity may also partly explain the non-normal distribution of several imaging parameters. Given the modest CMD sample size in this study (n = 36), further stratification into subgroups such as “isolated high IMR”, “isolated low CFR”, and “combined abnormalities” was not possible due to inadequate statistical power. Future multicenter studies with larger cohorts are therefore needed to perform sensitivity analyses across these endotypes, and to determine whether distinct microvascular phenotypes drive different structural or functional remodeling patterns.

## 6. Conclusions

In patients with INOCA, CMD was independently associated with CCTA-derived imaging features of early left-heart remodeling, particularly myocardial hypertrophy, eccentric LV remodeling, and left atrial enlargement. CCTA-derived LAVi and related volumetric indices may reflect early diastolic-related functional remodeling; however, these functional implications remain exploratory and require validation against established diastolic reference standards. Future multicenter prospective studies that integrate multimodal imaging and biomarkers are warranted to refine CMD endotype classification (structural vs. functional), clarify the temporal relationship between CMD and remodeling patterns, and further define the clinical value of CCTA for risk stratification and for guiding diagnostic evaluation.

## Data Availability

All raw data and code are available upon request.
