# Supplementary material for: Analysis of Cardiac Computed Tomography: Investigating the Relationship Between Coronary Microvascular Dysfunction and Left Heart Remodeling in Patients With Myocardial Ischemia Due to Non-Obstructive Coronary Artery Disease
Source: Rev Cardiovasc Med. 2026 Jul 17;27(7):49529. doi: 10.31083/RCM49529 (PMC13419969; doi:10.31083/RCM49529)
Supplement: Supplementary file 1 [file 2153-8174-27-7-49529-s1.zip › Supplementary 1-Supplementary Methods.docx]

**Supplementary Methods. Diagnostic checks for the primary logistic regression models**

In the primary analysis, CMD status was modeled as the dependent variable using binary logistic regression. Because the number of CMD events was modest (36 events) and the CCTA-derived remodeling parameters were biologically and statistically interrelated, we did not enter all CT parameters into a single multivariable model. Instead, each CT parameter was evaluated in a separate multivariable model with prespecified clinical covariates (age, sex, and hypertension) to reduce overfitting and improve model stability.

Multicollinearity among predictors included in each logistic regression model was assessed using variance inflation factors (VIFs), tolerance values, and collinearity diagnostics. Across the primary models, all VIF values were low, ranging from 1.030 to 1.354, indicating no substantial multicollinearity among the included predictors. The maximum condition index ranged from 19.453 to 21.198 across models, which did not suggest severe collinearity. Overall, these findings indicate that multicollinearity was unlikely to materially affect the stability of the regression estimates. These diagnostics supported the adequacy of the primary logistic regression framework used in the revised manuscript.
