# Supplementary material for: Analysis of Cardiac Computed Tomography: Investigating the Relationship Between Coronary Microvascular Dysfunction and Left Heart Remodeling in Patients With Myocardial Ischemia Due to Non-Obstructive Coronary Artery Disease
Source: Rev Cardiovasc Med. 2026 Jul 17;27(7):49529. doi: 10.31083/RCM49529 (PMC13419969; doi:10.31083/RCM49529)
Supplement: Supplementary file 1 [file 2153-8174-27-7-49529-s1.zip › Supplementary Table 1.docx]

**Supplementary 1**

**Table 1. Sensitivity logistic regression analyses of indexed CT parameters with additional adjustment for BMI**

| Variables | Primary multivariable | |  | BMI-adjusted | | |
| --- | --- | --- | --- | --- | --- | --- |
|  | OR (95%CI) | *P* |  | OR (95%CI) |  | *P* |
| LAMSVi | 1.129(1.052-1.211) | ＜0.001* |  | 1.129(1.052-1.212) |  | ＜0.001* |
| LAMDVi | 1.133(1.048-1.225) | 0.002 |  | 1.133(1.048-1.226) |  | 0.002 |
| LVMi | 1.072(1.020-1.127) | 0.006 |  | 1.073(1.020-1.129) |  | 0.006 |
| LVMDVi | 1.069(1.015-1.126) | 0.012 |  | 1.070(1.016-1.127) |  | 0.011 |
| LVMSVi | 1.084(1.007-1.168) | 0.032 |  | 1.087(1.008-1.171) |  | 0.030 |

The primary multivariable model was adjusted for age, sex, and hypertension. The sensitivity model additionally included BMI. Each CT parameter was entered separately into the multivariable logistic regression model together with the covariates.
