# Supplementary material for: Analysis of Cardiac Computed Tomography: Investigating the Relationship Between Coronary Microvascular Dysfunction and Left Heart Remodeling in Patients With Myocardial Ischemia Due to Non-Obstructive Coronary Artery Disease
Source: Rev Cardiovasc Med. 2026 Jul 17;27(7):49529. doi: 10.31083/RCM49529 (PMC13419969; doi:10.31083/RCM49529)
Supplement: Supplementary file 1 [file 2153-8174-27-7-49529-s1.zip › Supplementary Table 2.docx]

| Dependent variable | Non-CMD | CMD | Covariate | Adjusted difference  (CMD vs Non-CMD) | 95% CI | *P* |
| --- | --- | --- | --- | --- | --- | --- |
| Raw LVM (g) | 103.80 (91.80, 131.55) | 113.55 (99.38, 138.90) | BSA | 12.829 | 2.889, 22.769 | 0.012 |
| Raw LVMDV (ml) | 89.79 ± 24.26 | 99.28 ± 21.56 | BSA | 11.620 | 2.040, 21.200 | 0.018 |

Supplementary Table 2. ANCOVA of raw LVMDV and raw LVM by CMD status with BSA as a covariate

Data are presented as median (interquartile range) for non-normally distributed variables and mean ± standard deviation for normally distributed variables. Adjusted differences were estimated from ANCOVA models with CMD status as the fixed factor and BSA as a covariate. Positive adjusted differences indicate higher values in the CMD group.
