# Supplementary material for: Analysis of Cardiac Computed Tomography: Investigating the Relationship Between Coronary Microvascular Dysfunction and Left Heart Remodeling in Patients With Myocardial Ischemia Due to Non-Obstructive Coronary Artery Disease
Source: Rev Cardiovasc Med. 2026 Jul 17;27(7):49529. doi: 10.31083/RCM49529 (PMC13419969; doi:10.31083/RCM49529)
Supplement: Supplementary file 1 [file 2153-8174-27-7-49529-s1.zip › Supplementary Table 3.docx]

| Predictor | Adjusted covariates | B | SE | OR [Exp(B)] | 95% CI | *P* |
| --- | --- | --- | --- | --- | --- | --- |
| Raw LVM | BSA, Age, Sex, Hypertension | 0.038 | 0.014 | 1.039 | 1.010, 1.069 | 0.007 |
| Raw LVMDV | BSA, Age, Sex, Hypertension | 0.037 | 0.015 | 1.037 | 1.007, 1.068 | 0.015 |

Supplementary Table 3. Sensitivity logistic regression analyses using raw LVM and raw LVMDV with adjustment for BSA

CMD was the dependent variable. Sensitivity logistic regression models were fitted using raw, non-indexed CT parameters as predictors, with adjustment for BSA, age, sex, and hypertension.
