# Supplementary material for: Analysis of Cardiac Computed Tomography: Investigating the Relationship Between Coronary Microvascular Dysfunction and Left Heart Remodeling in Patients With Myocardial Ischemia Due to Non-Obstructive Coronary Artery Disease
Source: Rev Cardiovasc Med. 2026 Jul 17;27(7):49529. doi: 10.31083/RCM49529 (PMC13419969; doi:10.31083/RCM49529)
Supplement: Supplementary file 1 [file 2153-8174-27-7-49529-s1.zip › Supplementary Table 4.docx]

Supplementary Table 4. Sensitivity analysis using an alternative CMD definition (IMR >25 or CFR <2.5): group comparisons of key CCTA remodeling indices

| Variables | Total | Non-CMD | CMD | Statistic | *P* | 95% CI | Effect Size |
| --- | --- | --- | --- | --- | --- | --- | --- |
|  | n = 74 | n = 36 | n = 38 |  |  |  |  |
| LVMi (g/m^2^) | 65.96 ± 12.94 | 62.86 ± 11.84 | 68.90 ± 13.40 | t=-2.05 | 0.044* | -11.911, -1.689 | 0.475 |
| LVMDVi (ml/m^2^) | 54.20 ± 12.19 | 51.90 ± 11.27 | 56.37 ± 12.76 | t=-1.60 | 0.115 | -10.069, 1.112 | 0.339 |
| LVMSVi (ml/m^2^) | 22.41 (17.88, 28.27) | 20.68 (16.43, 25.87) | 23.13 (19.81, 29.63) | Z=-1.17 | 0.077 | -6.223, 0.498 | 0.278 |
| LAMDVi (ml/m^2^) | 45.21 (41.08, 50.60) | 42.70 (38.70, 46.88) | 48.40 (43.18, 53.63) | Z=-3.28 | 0.001* | -10.364, -2.616 | 0.778 |
| LAMSVi (ml/m^2^) | 55.40 ± 11.55 | 50.72 ± 9.30 | 59.84 ± 11.82 | t=-3.68 | ＜0.001* | -14.070, -4.177 | 0.847 |
