# Supplementary material for: Analysis of Cardiac Computed Tomography: Investigating the Relationship Between Coronary Microvascular Dysfunction and Left Heart Remodeling in Patients With Myocardial Ischemia Due to Non-Obstructive Coronary Artery Disease
Source: Rev Cardiovasc Med. 2026 Jul 17;27(7):49529. doi: 10.31083/RCM49529 (PMC13419969; doi:10.31083/RCM49529)
Supplement: Supplementary file 1 [file 2153-8174-27-7-49529-s1.zip › Supplementary Table 5.docx]

Supplementary Table 5. Sensitivity logistic regression with alternative CMD definition (IMR＞25 or CFR＜ 2.5).

| Variables | Univariate | |  | Multivariate | | |
| --- | --- | --- | --- | --- | --- | --- |
|  | OR (95%CI) | *P* |  | OR (95%CI) |  | *P* |
| LAMSVi | 1.102(1.036-1.172) | 0.002 |  | 1.108(1.039-1.182) |  | 0.002 |
| LAMDVi | 1.112(1.037-1.193) | 0.003 |  | 1.133(1.048-1.225) |  | 0.002 |
| LVMi | 1.040(1.000-1.081) | 0.049 |  | 1.067(1.015-1.121) |  | 0.010 |
| LVMDVi | 1.033(0.992-1.075) | 0.119 |  | 1.048(1.000-1.099) |  | 0.049 |
| LVMSVi | 1.055(0.990-1.124) | 0.098 |  | 1.071(0.996-1.150) |  | 0.063 |

Multivariable models were adjusted for age, sex, and hypertension, consistent with the primary analysis shown in Table 4.
